# Circulating exosomal mRNA signatures for the early diagnosis of clear cell renal cell carcinoma

**DOI:** 10.1186/s12916-022-02467-1

**Published:** 2022-08-25

**Authors:** Xing He, Feng Tian, Fei Guo, Fangxing Zhang, Huiyong Zhang, Jin Ji, Lin Zhao, Jingyi He, Yutian Xiao, Longman Li, Chunmeng Wei, Caihong Huang, Yexin Li, Feng Zhang, Bo Yang, Huamao Ye, Fubo Wang

**Affiliations:** 1 Department of Urology, Changhai Hospital, Naval Medical University (Second Military Medical University), 168 Changhai Road, Shanghai, 200433 China; 2 Department of Urology, The Eighth People’s Hospital of Shanghai, 8 Caobao Road, Shanghai, 200235 China; 3 Center for Genomic and Personalized Medicine, Guangxi Key Laboratory for Genomic and Personalized Medicine, Guangxi Collaborative Innovation Center for Genomic and Personalized Medicine, Guangxi Medical University, 22 Shuangyong Road, Nanning, 530021 Guangxi China

**Keywords:** Clear cell renal cell carcinoma, Exosome, mRNA, Signature, Diagnosis

## Abstract

**Background:**

There are no proven tumor biomarkers for the early diagnosis of clear cell renal cell carcinoma (ccRCC) thus far. This study aimed to identify novel biomarkers of ccRCC based on exosomal mRNA (emRNA) profiling and develop emRNA-based signatures for the early detection of ccRCC.

**Methods:**

Four hundred eighty-eight participants, including 226 localized ccRCCs, 73 patients with benign renal masses, and 189 healthy controls, were recruited. Circulating emRNA sequencing was performed in 12 ccRCCs and 22 healthy controls in the discovery phase. The candidate emRNAs were evaluated with 108 ccRCCs and 70 healthy controls in the test and training phases. The emRNA-based signatures were developed by logistic regression analysis and validated with additional cohorts of 106 ccRCCs, 97 healthy controls, and 73 benign individuals.

**Results:**

Five emRNAs, CUL9, KMT2D, PBRM1, PREX2, and SETD2, were identified as novel potential biomarkers of ccRCC. We further developed an early diagnostic signature that comprised KMT2D and PREX2 and a differential diagnostic signature that comprised CUL9, KMT2D, and PREX2 for RCC detection. The early diagnostic signature displayed high accuracy in distinguishing ccRCCs from healthy controls, with areas under the receiver operating characteristic curve (AUCs) of 0.836 and 0.830 in the training and validation cohorts, respectively. The differential diagnostic signature also showed great performance in distinguishing ccRCCs from benign renal masses (AUC = 0.816), including solid masses (AUC = 0.810) and cystic masses (AUC = 0.832).

**Conclusions:**

We established and validated novel emRNA-based signatures for the early detection of ccRCC and differential diagnosis of uncertain renal masses. These signatures could be promising and noninvasive biomarkers for ccRCC detection and thus improve the prognosis of ccRCC patients.

**Supplementary Information:**

The online version contains supplementary material available at 10.1186/s12916-022-02467-1.

## Highlights

1. Circulating exosomal RNA sequencing identified novel potential biomarkers of clear cell renal cell carcinoma (ccRCC).

2. The early diagnostic signature comprising KMT2D and PREX2 showed high accuracy in distinguishing ccRCCs from healthy individuals.

3. The differential diagnostic signature comprising CUL9, KMT2D, and PREX2 showed great performance in distinguishing ccRCCs from benign renal masses.

4. These signatures could be promising and noninvasive biomarkers for ccRCC detection.

**Supplementary Information:**

The online version contains supplementary material available at 10.1186/s12916-022-02467-1.

## Background

Renal cell carcinoma (RCC) originates from renal tubular epithelial cells, accounting for more than 90% of malignant renal tumors [1]. The incidence of RCC is increasing steadily worldwide, with a higher increasing rate in developing countries and a higher incidence rate in developed countries [2]. Early RCC is organ-confined with a 5-year survival rate of over 90%. However, once RCC invades local organs or spreads distantly, the treatment of the tumor is quite complex, and the prognosis is poor [3]. Clear cell RCC (ccRCC) is the most common subtype, accounting for about 75% of newly diagnosed RCC [1]. Therefore, the accurate early detection of ccRCC is of great importance for the management of RCC. Unfortunately, there are no proven tumor biomarkers for the early diagnosis of ccRCC thus far.

Exosomes are small extracellular vesicles 40–150 nm in diameter secreted by cells that contain molecules such as nucleic acids, proteins, lipids, amino acids, and metabolites, and they play an important role in cellular communication and regulating the physiological and pathological processes of the human body [4]. More importantly, the lipid bilayer membrane structure of exosomes is resistant to the effect of exogenous proteases and RNA enzymes, leading to more stable substances, such as messenger RNAs (mRNAs), microRNAs (miRNAs), and functional proteins [5, 6]. Tumor-derived exosomes carrying various molecules could provide a promising noninvasive method for detecting cancers [7].

Recently, emerging reports have indicated that circulating exosomal RNAs (exRNAs) can serve as promising biomarkers for cancer detection [8–10]. Studies have shown that exosomal miRNAs in blood and urine could effectively discriminate renal cell carcinoma from healthy controls, thus suggesting that exosomal miRNAs could be used as biomarkers to detect RCC [11, 12]. Owing to the broad adoption of small RNA sequencing by ERCC1 groups and researchers’ recent exploration, other RNA biotypes, especially long RNAs, have been examined [13]. Recent studies have shown that extracellular vesicles long RNAs (exLRs), mainly mRNAs, could be potential biomarkers for prostate cancer (PCa) [14], glioma [15], hepatocellular carcinoma (HCC) [16, 17], pancreatic ductal adenocarcinoma (PDAC) [18], etc. Although some preliminary studies have been published trying to identify emRNAs for detecting ccRCC, their clinical performance has largely been limited because of the study design (i.e., small sample size [19], lack of a true negative control [19], and lack of validation in clinical samples [20]).

In this study, we systematically investigated circulating exosomal mRNA (emRNA) profiling by RNA sequencing between localized ccRCCs and healthy controls and found a number of ccRCC-associated emRNAs. We then identified and validated several emRNAs with diagnostic potential in a large cohort of 488 participants. Finally, we established emRNA-based signatures for the early diagnosis of ccRCC.

## Results

### Patients’ characteristics

The demographic and clinical characteristics of the discovery, test, training, and validation cohorts of participants are presented in Table 1 and Additional file 2: Table S1. There was no significant difference in the distribution of sex and age between localized ccRCCs and healthy controls in the discovery, test, training, and validation cohorts of participants (Table 1). For participants in the ccRCC group and patients with benign masses group in the validation cohort, the distribution of age between ccRCCs and benign controls was similar. The mean baseline tumor sizes were all less than 4 cm in four cohorts of localized ccRCCs. The clinical tumor stages were T1-2 in four cohorts of localized ccRCCs. In the vast majority of ccRCC cases, the pathological grade was G1-2 (83.3% in the discovery cohort, 93.8% in the test cohort, 88.0% in the training cohort, 96.2% in the validation cohort). The demographic and clinical characteristics of participants with benign solid and cystic masses group are presented in Additional file 2: Table S1. There were fewer male participants in the solid group, and the participants in the solid group were younger than those in the cystic group and ccRCC group. The tumor sizes were larger in the cystic group than in the solid group.

**Table 1:** Demographic and clinical characteristics of the discovery, test, training, and validation cohorts of participants

Characteristics	Discovery set			Test set			Training set			Validation set				
	**ccRCC**	**Healthy**	***p***	**ccRCC**	**Healthy**	***p***	**ccRCC**	**Healthy**	***p***	**ccRCC**	**Healthy**	***p***	**Benign**	***p***
	***n* = 12**	***n* = 22**		***n* = 16**	***n* = 20**		***n* = 92**	***n* = 50**		***n* = 106**	***n* = 97**		***n* = 73**	
**Age (years)**			0.500			0.438			0.062			0.125		0.176
Mean ± SD	55.6 ± 15.4	52.1 ± 13.9		52.4 ± 11.6	55.9 ± 13.9		56.2 ± 11.8	59.8 ± 8.4		54.0 ± 11.0	55.9 ± 5.5		51.6 ± 12.7	
**Sex, *n* (%)**			0.297			0.157			0.153			0.294		0.000
Male	8 (66.7)	10 (45.5)		13 (81.3)	11 (55.0)		66 (71.7)	30 (60.0)		73 (68.9)	60 (61.9)		30 (41.1)	
Female	4 (33.3)	12 (54.5)		3 (18.7)	9 (45.0)		26 (28.3)	20 (40.0)		33 (31.1)	37 (38.1)		43 (58.9)	
**Tumor size (cm)**														
Mean ± SD	3.8 ± 2.2			3.1 ± 1.5			3. 5 ± 1.6			3.6 ± 1.8			4.8 ± 2.8	
**Tumor stage, *n* (%)**														
T1a	10 (83.3)			13 (81.3)			69 (75.0)			77 (72.6)				
T1b	2 (16.7)			3 (18.7)			19 (20.7)			24 (22.6)				
T2	0			0			4 (4.3)			5 (4.7)				
**Pathological grade (WHO/ISUP), *n* (%)**														
1	1 (8.3)			1 (6.3)			6 (6.5)			10 (9.4)				
2	9 (75.0)			14 (87.5)			75 (81.5)			92 (86.8)				
3	1(8.3)			1 (6.3)			10 (10.9)			4 (3.8)				
4	0			0			1 (1.1)			0				
Unclassified	1 (8.3)			0			0			0				

*Abbreviation*: *ccRCC*, clear cell renal cell carcinoma

### Circulating exosomal RNA screening and testing

In the discovery phase, we investigated circulating emRNA profiling between 12 localized ccRCCs (*n* = 12) and healthy controls (*n* = 22) by RNA-seq. Then, based on the RNA sequencing data, 210 dysregulated emRNAs (*p* < 0.05, fold change > 2 or < 0.5, FDR < 0.05, Additional file 2: Table S3) were identified. The heatmap illustrated the expression levels of the representative ccRCC-associated emRNAs (Additional file 1: Fig. S2A). Seven top upregulated emRNAs and related to ccRCC or/and multiple malignant tumors, including CUL9, ATM, ARID1A, KMT2D, PBRM1, PREX2, and SETD2, were selected as candidate biomarkers for further testing. Then, we tested the expression levels of these seven candidate emRNAs by RT–qPCR in an additional cohort of localized ccRCCs (*n* = 16) and healthy controls (*n* = 20). ATM and ARID1A were excluded because they showed no difference between the ccRCC group and the healthy group (Additional file 1: Fig. S2B). Finally, the remaining 5 emRNAs (CUL9, KMT2D, PBRM1, PREX2, and SETD2) that were upregulated in ccRCCs were included for training and validation (Additional file 1: Fig. S2B).

### Circulating candidate emRNA expression levels for ccRCCs versus healthy controls in the training and validation phases

**Fig. 1:**
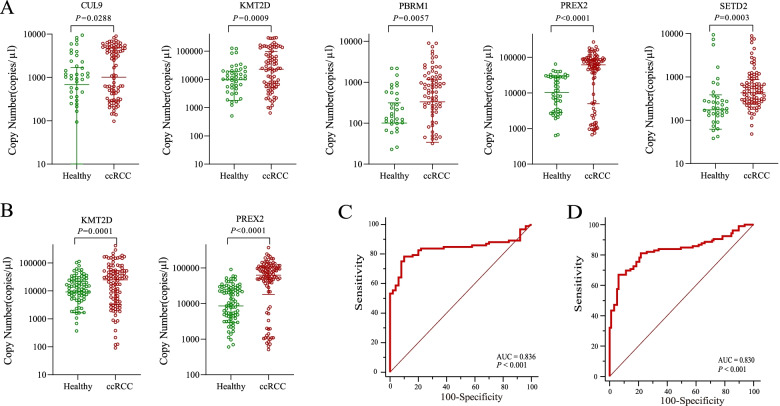
Validating ccRCC-associated circulating emRNAs and establishing an emRNA-based clear cell renal cell carcinoma (ccRCC) early diagnostic signature. **A** Scatter plots showing the expression levels of ccRCC-associated circulating emRNAs, including CUL9, KMT2D, PBRM1, PREX2, and SETD2, between localized ccRCCs (*n* = 92) and healthy controls (*n* = 50) in the training phase. **B** Scatter plots showing the expression levels of KMT2D and PREX2 identified as significant biomarkers for ccRCC diagnosis by multivariate logistic regression analysis between localized ccRCCs (*n* = 106) and healthy controls (*n* = 97) in the validation phase. **C**,** D** ROC-AUC evaluation showed the diagnostic performance of the signature comprising KMT2D and PREX2 to distinguish localized ccRCCs from healthy controls in the training phase (*n* = 142) and validation phase (*n* = 203). ccRCC, clear cell renal cell carcinoma; ROC, receiver operator characteristic; AUC, area under the curve

In the training phase, the expression levels of emRNAs were detected in an additional cohort of 142 clinical samples, including localized ccRCCs (*n* = 92) and healthy controls (*n* = 50), by RT–qPCR, which revealed that CUL9, KMT2D, PBRM1, PREX2, and SETD2 were significantly higher in the ccRCC group than in the healthy group, and each of the candidate biomarkers achieved good performance for distinguishing localized ccRCCs from healthy controls with corresponding AUCs of 0.611, 0.668, 0.639, 0.742, and 0.680, respectively (Table 2, Fig. 1A, and Additional file 1: Fig. S3A). Furthermore, KMT2D and PREX2 were further identified as significant biomarkers for ccRCC diagnosis by multivariate logistic regression analysis (Table 2). These two emRNAs were then validated in an additional cohort of localized ccRCCs (*n* = 106) and healthy controls (*n* = 97). High expression levels of KMT2D and PREX2 were observed in the ccRCC group compared to the healthy group (Fig. 1B). The diagnostic accuracy of KMT2D and PREX2 had AUCs of 0.655 and 0.776, respectively (Table 2, Additional file 1: Fig. S3B). After multivariate logistic regression analysis, KMT2D and PREX2 still displayed statistical significance for distinguishing localized ccRCCs from controls (Table 2).

**Table 2:** Diagnostic performance of candidate emRNAs for distinguishing localized clear cell renal cell carcinoma (ccRCC) patients from healthy controls in the training and validation sets

mRNA group	Training set				Validation set			
	**Univariate**			**Multivariate**	**Univariate**			**Multivariate**
	***p***	**Youden index**	**AUC**	***p***	***p***	**Youden index**	**AUC**	***p***
CUL9	0.0266	0.27	0.611	/	/	/	/	/
KMT2D	0.0002	0.35	0.668	0.0011	< 0.0001	0.29	0.655	0.0013
PBRM1	0.0029	0.30	0.639	/	/	/	/	/
PREX2	< 0.0001	0.62	0.742	< 0.0001	< 0.0001	0.61	0.776	< 0.0001
SETD2	0.0007	0.41	0.680	/	/	/	/	/

Note. AUC of ccRCC diagnostic signature for ccRCC versus healthy = 0.836 (95% CI, 0.765 to 0.893). Logistic regression model (method of stepwise) = 1.21943 × KMT2D + 1.97072 × PREX2 + 1.30485. *ccRCC*, clear cell renal cell carcinoma; *ROC*, receiver operator characteristic; *AUC*, area under the curve

### Establishing an emRNA-based RCC signature for distinguishing ccRCCs from healthy controls

In the training cohorts (*n* = 142), we used multivariate logistic regression analysis to establish an emRNA-based ccRCC signature comprising KMT2D and PREX2. The diagnostic performance of the signature (logit (*p* = ccRCC) = 1.21943 × KMT2D + 1.97072 × PREX2 + 1.30485), measured by AUC, was 0.836 (*p* < 0.001; 95% CI, 0.765 to 0.893, Fig. 1C). The novel biomarkers (KMT2D and PREX2) identified from the training set were applied in the validation cohort of participants (*n* = 203). Then, these genes were applied to the above model and the ROC was constructed. The diagnostic signature also displayed high accuracy in distinguishing ccRCCs from healthy controls, with an area under the receiver operating characteristic curve (AUC) of 0.830 (*p* < 0.001; 95% CI, 0.771 to 0.879, Fig. 1D). These results showed that the emRNA signature could serve as a novel method for the early detection of ccRCC from healthy controls.

### Establishing an emRNA-based RCC signature for distinguishing ccRCCs from benign renal masses

**Fig. 2:**
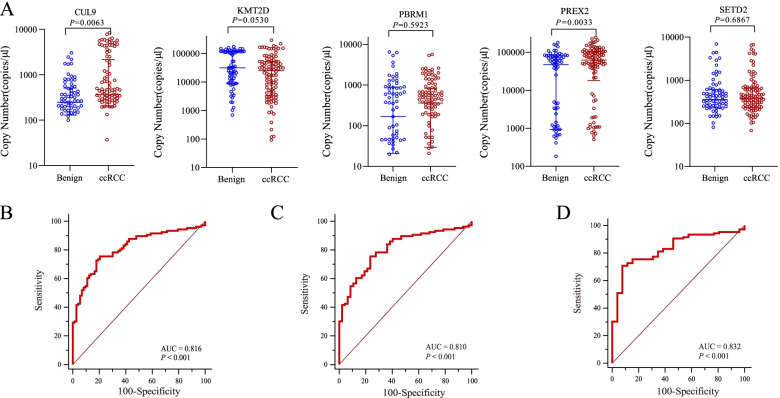
Establishing an emRNA-based clear cell renal cell carcinoma (ccRCC) diagnostic signature.** A** Scatter plots showing the expression levels of candidate emRNAs, including CUL9, KMT2D, PBRM1, PREX2, and SETD2, between localized ccRCCs (*n* = 106) and patients with benign renal masses (*n* = 73). **B** ROC-AUC evaluation showed the diagnostic performance of the diagnostic signature comprising CUL9, KMT2D, and PREX2 to differentiate localized ccRCCs (*n* = 106) from patients with benign renal masses (*n* = 73). **C**, **D** ROC-AUC evaluation showed the diagnostic performance of the signature comprising CUL9, KMT2D, and PREX2 to differentiate localized ccRCCs (*n* = 106) from patients with benign solid masses (*n* = 47) and benign cystic masses (*n* = 26). ccRCC, clear cell renal cell carcinoma; ROC, receiver operator characteristic; AUC, area under the curve

It is of great importance to diagnose ccRCC from a small renal mass. Therefore, we collected extra samples from participants with benign renal masses (*n* = 73), including solid masses (*n* = 47) and cystic masses (*n* = 26), to evaluate the diagnostic role of the 5 candidate emRNAs, including CUL9, KMT2D, PBRM1, PREX2, and SETD2, in distinguishing ccRCCs from patients with benign renal masses. We first evaluated the expression levels of candidate emRNAs between ccRCCs and patients with benign renal masses. High expression levels of CUL9 and PREX2 were observed in ccRCCs compared to those in patients with benign renal masses (Fig. 2A). The diagnostic power of CUL9, KMT2D, and PREX2 had AUCs of 0.619, 0.585, and 0.629, respectively (Table 3, Additional file 1: Fig. S3C). After multivariate logistic regression analysis, these three candidates still displayed statistical significance for distinguishing ccRCCs from patients with benign renal masses with multivariate *p* values < 0.0001, < 0.0001, and 0.0001, respectively (Table 3). To evaluate the performance of the signature derived to distinguish ccRCC from healthy controls in the differentiate diagnosis for renal masses, we applied the signature in differentiating ccRCCs from patients with benign renal masses. However, the diagnostic performance of the signature, evaluated by AUC, was only 0.559 (Additional file 1: Fig. S4), indicating that the signature for detecting ccRCC from healthy control was not applicable in differential diagnosing renal masses. We then used logistic regression analysis to establish an emRNA-based diagnostic signature using CUL9, KMT2D, and PREX2, logit (*p* = ccRCC) = 1.68689 × CUL9 − 1.16173 × KMT2D + 1.17881 × PREX2 + 0.75145. The signature showed great performance in diagnosing ccRCCs from patients with benign renal masses (AUC = 0.816, *p* < 0.001; 95% CI, 0.751 to 0.870, Fig. 2B). In addition, the diagnostic signature also showed excellent ability to distinguish ccRCC from benign solid masses (AUC = 0.810, *p* < 0.001; 95% CI, 0.739 to 0.869, Fig. 2C) and benign cystic masses (AUC = 0.832, *p* < 0.001; 95% CI, 0.757 to 0.891, Fig. 2D), respectively. These results indicated that the emRNA-based diagnostic signature could be a novel noninvasive biomarker for the improvement of ccRCC diagnosis.

**Table 3:** Diagnostic performance of candidate emRNAs for distinguishing localized clear cell renal cell carcinoma (ccRCC) patients from patients with benign renal masses

mRNA group	ccRCC vs benign				ccRCC vs benign (solid)	ccRCC vs benign (cystic)
	**Univariate**			**Multivariate**	**Multivariate**	**Multivariate**
	***p***	**Youden index**	**AUC**	***p***	***p***	***p***
CUL9	0.0039	0.23	0.619	< 0.0001	0.0005	0.0095
KMT2D	0.0504	0.25	0.585	< 0.0001	0.0010	0.0003
PBRM1	0.5989	0.16	0.524	/	/	/
PREX2	0.0022	0.24	0.629	0.0001	0.0003	0.0048
SETD2	0.6855	0.07	0.518	/	/	/

Note. AUC of ccRCC diagnostic signature for ccRCC versus benign = 0.816 (95% CI, 0.751 to 0.870). Logistic regression model (method of stepwise) = 1.68689 × CUL9 − 1.16173 × KMT2D + 1.17881 × PREX2 + 0.75145. *ccRCC*, clear cell renal cell carcinoma; *ROC*, receiver operator characteristic; *AUC*, area under the curve

## Discussion

As one of the most dangerous urological malignancies, ccRCC still does not have ideal diagnostic tools or strategies. A multicenter prospective observational cohort study of 706 patients in the UK expounded the importance of early detection of RCC, suggesting that focusing on RCC-related symptoms is limited to improving the early diagnosis of RCC [21]. More attention should be given to the improvement of diagnostic strategies and the identification of diagnostic biomarkers.

Approximately 60% of RCC patients are diagnosed incidentally, and most of them are diagnosed by imaging examination [21]. Ultrasound, as a relatively cheap, convenient, and safe examination, is able to detect 85–100% of tumors > 3 cm in size but only 67–82% of tumors 2–3 cm in size, suggesting that there may be more false-negative results in the detection of tumors < 3 cm in size using ultrasound [22]. On the other hand, computed tomography (CT) and magnetic resonance imaging (MRI) are more sensitive and specific than ultrasound for the detection of RCC. However, due to the low cost-effectiveness and radiation dose, it is unlikely that CT or MRI should be recommended for population screening [23]. Moreover, CT or MRI examination often detects some other incidental findings that may lead to overdiagnosis of a variety of uncertain visceral masses. Therefore, creating a cost-effective, accurate, feasible, and low-risk screening modality for ccRCC should be taken into account.

Currently, an increasing number of studies are dedicated to the discovery of diagnostic biomarkers for ccRCC. Several biomarkers found in serum and urine have been recommended as novel screening or diagnostic tools that are able to differentiate RCC from nonrenal urological tumors, benign renal masses, and healthy controls [24]. Researchers found that urine AQP1 and PLIN2 were significantly upregulated in patients with ccRCC and papillary RCC (*n* = 47), showing high potential as screening biomarkers for RCC and a differential diagnostic tool for renal masses [25]. However, due to the small sample size, urine AQP1 and PLIN2 need to be further validated. Another group of researchers developed a serum/plasma three-marker assay composed of N-methyltransferase (NNMT), L-plastin (LCP1), and nonmetastatic cells 1 protein (NM23A) to improve the early detection of RCC. The three-marker assay has high sensitivity, specificity, and diagnostic AUC (95.6%, 90.9%, and 0.932) for RCC compared to healthy controls and benign tumors, but has limited ability to differentiate RCC from benign renal masses [26]. In general, liquid biopsy using blood or urine still has huge potential for improving the early detection and differential diagnosis of RCC.

The characteristics of exosomes make exosome-related assays an increasingly noteworthy noninvasive and effective method for disease diagnosis and monitoring [8, 27]. The strong potential of exosomes in diagnosis and treatment has been proven in several types of cancer, such as prostate, bladder, and breast cancer [28, 29]. Currently, studies have found that exosomal RNAs, especially miRNAs, may have great potential in the detection of RCC [30]. MiR‐21 levels were higher in ccRCCs than in healthy controls in both sera and serum exosomes. Serum exosomal miR‐210 could effectively distinguish ccRCCs from controls (AUC = 0.8779) [31]. Another study found that serum exosomal miR-210 and miR-1233 could be novel biomarkers to diagnose ccRCC [32]. Others found that hsa-mir-149-3p and hsa-mir-424-3p discovered by exosomal miRNA sequencing had higher expression levels in RCCs than in healthy controls [12]. However, these previous studies focused on circulating miRNAs, and there are still no studies exploring the potential role of circulating emRNAs in the early detection of ccRCC. In this study, we systematically investigated circulating emRNA profiling by RNA sequencing between localized ccRCCs and healthy controls and found a number of ccRCC-associated emRNAs. We then identified 5 ccRCC-associated emRNAs with diagnostic potential and established an emRNA-based screening signature with great ability for ccRCC detection. This signature could serve as a new biomarker to screen out ccRCC from the normal population.

With the widespread use of cross-sectional imaging, the incidence of renal masses, especially small renal masses (SRMs, diameter < 4 cm), has displayed a significant increase [33]. SRMs raise the difficulty of differential diagnosis. It has been reported that 20% of renal masses < 4 cm were identified as benign histology, while the proportion of benign histology in those ≥ 7 cm was 6.3% [34]. Renal masses can be divided into solid masses, cystic masses, and inflammatory masses [35]. The majority of patients with renal masses are asymptomatic, and the main morphology of the masses is solid and cystic. The most common benign solid renal mass in clinical practice is angiomyolipoma (AML) [36, 37]. However, nearly 5% of AMLs, called lipid-poor AML, have too little fat to be diagnosed by CT or MRI [38, 39]. Therefore, lipid-poor AMLs are difficult to distinguish from RCC, and some of these tumors are diagnosed after surgery [40]. Renal oncocytoma (RO) is a benign epithelial neoplasm usually containing large cells with granular eosinophilic cytoplasm [41]. However, perinephric fat invasion occurs in 2–20% of RO cases, and approximately 5.4% of RO cases display vascular invasion [42]. These features are commonly considered a characteristic of malignancy in RCC, which may increase the difficulty of differential diagnosis. On the other hand, most renal cystic masses are nonneoplastic cystic, but many renal tumors contain cysts as a minor or dominant component; thus, the differential diagnosis of benign and malignant renal cystic masses is necessary [43]. To identify ccRCC, it is important to distinguish renal malignant masses from benign solid and cystic masses. Developing a noninvasive method of differential diagnosis remains of great potential value in clinical practice.

In our study, we further collected the serum from 106 ccRCCs and 73 patients with benign renal masses and then constructed a ccRCC diagnostic signature with three candidate genes that were screened out by multivariate stepwise logistic regression analysis. This signature displayed an excellent ability to distinguish ccRCCs from patients with benign renal masses, whether solid or cystic. Therefore, the diagnostic signatures could distinguish those with an uncertain nature of renal masses, such as cystic renal carcinoma, fat-poor angiomyolipoma, and renal oncocytoma, especially if the mass was small and the essence of the tumor could not be determined by imaging.

Our study also had a few limitations. Firstly, as a single-center retrospective study, the sample size was limited. The findings, especially the performance of the emRNA signature for diagnosing renal masses, need to be validated in a further multicenter prospective large-scale study. Secondly, due to insufficient samples for other types of renal cystic masses, our study could not make a differential diagnosis for Bosniak-graded renal masses. Finally, this study mainly focused on the exploration of biomarkers in peripheral blood suitable for early diagnosis of tumors, but did not include the analysis of clinical factors such as smoking, obesity, hypertension, and other variables, which could be combined with clinical characteristics to establish a comprehensive diagnostic and prognostic model of tumors in the follow-up study. Cellular and animal experiments were also not conducted, lacking certain theoretical support, and the role and secretion mechanism of exosomal mRNA and RCC will continue to be explored subsequently.

## Conclusions

We reported a systematic ccRCC biomarker investigation using circulating emRNA profiling for the first time. We established and validated novel emRNA-based signatures for the early detection of ccRCC and differential diagnosis of uncertain renal masses. These signatures could be promising and noninvasive biomarkers for ccRCC detection and thus improve the prognosis of ccRCC patients.

## Methods

### Participants

This study was approved by the Clinical Research Ethics Committee of Shanghai Changhai Hospital (Shanghai, China) (no. CHEC2020-112). All of the clinical samples were obtained from Shanghai Changhai Hospital. Written informed consent was obtained from the participants before sampling. In this study, 488 participants, including localized ccRCCs (*n* = 226), patients with benign renal masses (*n* = 73), and healthy controls (*n* = 189), were recruited consecutively from August 2017 to July 2020. The renal masses were confirmed by surgical pathology and examined by two independent pathologists. Tumor stage and pathological grade were estimated according to the 8th TNM criteria proposed by the American Joint Committee on Cancer (AJCC) in 2017. Inclusion criteria for patients with renal masses were as follows: (1) age 20–80 years old; (2) CT scans prior to surgery; (3) a definite diagnosis by pathology; (4) ccRCC participants with clinical tumor stages I and II (localized renal carcinoma); (5) received surgical operation, did not undergo any anticancer treatment before sampling; (6) no history of other cancers; and (7) signed informed consent. Inclusion criteria for healthy controls were as follows: (1) age 20–80 years old, (2) underwent a health check-up and considered asymptomatic and healthy, (3) ultrasonic examination showed no mass, and (4) signed informed consent. The exclusion criteria were as follows: (1) previously diagnosed renal masses, (2) received ablation therapy, and (3) with other malignant tumors.

### Study design

**Fig. 3:**
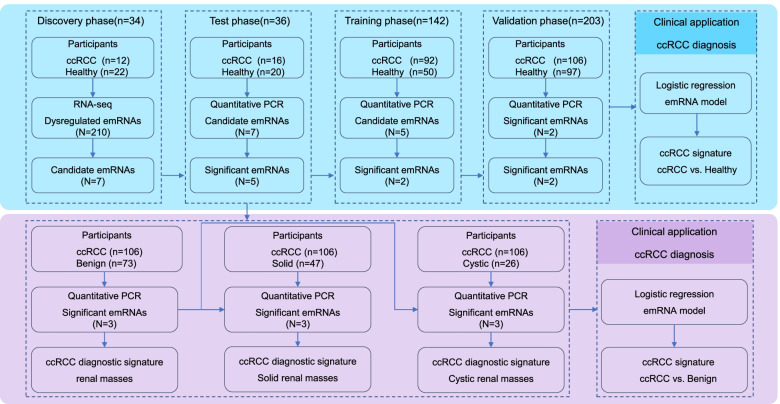
Workflow of the study design. ccRCC, clear cell renal cell carcinoma; RNA-seq, RNA sequencing; emRNA, exosomal messenger RNA; PCR, polymerase chain reaction

This study consisted of four phases: discovery, test, training, and validation. The participants in the discovery, training, and validation phases were consecutively enrolled, and they were randomly assigned into these groups. The participants in the test phase were randomly selected from the training phase. An overview of the workflow is summarized in Fig. 3. The clinical characteristics of the participants are summarized in Table 1. In the discovery phase, we investigated circulating emRNA profiling in ccRCCs (*n* = 12) and healthy controls (*n* = 22) by RNA-seq. Dysregulated (*p* < 0.05, fold change > 2 or < 0.5, FDR < 0.05) emRNAs in ccRCC were identified. The seven top upregulated emRNAs that were also related to ccRCC or/and multiple malignant tumors were selected as candidate biomarkers for further training and validation (see details of ‘The selection strategy of candidate biomarkers’ in Additional file 3: Supporting information). In the test phase, the expression levels of the candidate emRNAs were evaluated in localized ccRCCs (*n* = 16) and healthy controls (*n* = 20) by RT–qPCR. In the training phase, the expression levels of the significant emRNAs identified in the test phase were evaluated in another cohort of localized ccRCCs (*n* = 92) and healthy controls (*n* = 50) by RT–qPCR. A stepwise logistic regression analysis was used to identify the significant predictors and establish an emRNA-based ccRCC signature to differentiate ccRCCs from healthy controls. The signature was then validated in an additional cohort of localized ccRCCs (*n* = 106) and healthy controls (*n* = 97). In addition, we evaluated the diagnostic performance of the candidate emRNAs in distinguishing ccRCCs from patients with benign renal masses. In this phase, patients with benign renal masses (*n* = 73), including solid renal masses (*n* = 47) and cystic renal masses (*n* = 26), were included (the detailed clinical characteristics are summarized in Additional file 2: Table S1). Likewise, we applied stepwise logistic regression analysis to identify the significant predictors and establish an emRNA-based signature to differentiate ccRCCs from patients with benign renal masses, including solid and cystic masses.

### Sample collection and processing

The patients with renal masses signed informed consent on the first day of admission, and their fasting peripheral blood was collected on the second morning. Healthy controls signed informed consent on the day of health check-up and their fasting peripheral blood was collected before the physical examination. Samples were stored in a 4 °C refrigerator and transported to the laboratory with ice. Blood samples were allowed to clot at room temperature for a minimum of 30 min and a maximum of 2 h. All samples were then centrifuged at 1600 × g for 15 min to separate the serum, and the serum (supernatant) was collected in 1.5-ml centrifuge tubes and numbered. The serum samples were immediately stored at − 80 °C until further processing.

### Exosome isolation

An exoEasy Maxi Kit (No. 76064, Qiagen, Dusseldorf, North Rhine-Westphalia, Germany) was used to purify exosomes from serum according to the manufacturer’s instructions. First, the serum was filtered through a 0.22-μm filter membrane, an equal volume of XBP buffer wad added, and then it was gently inverted and mixed 5 times. After mixing, the above sample mixture was added to the adsorption column and centrifuged at 500 × g for 1 min at room temperature. The flow-through solution was discarded from the adsorption column. This procedure was repeated once. Then, approximately 5 ml of XWP buffer was added to the adsorption column for cleaning and centrifuged at 5000 × g for 5 min at room temperature. The adsorption column was discarded, and the serum exosomes were immobilized on the adsorption column membrane. After that, the adsorption column was placed into a new collection tube, and 400 μl XE buffer was added to the column for elution, then incubated at room temperature for 1 min, and centrifuged at 500 × g for 5 min. Finally, the eluate in the collection tube was added back into the adsorption column and incubated for 1 min at room temperature. The eluate was centrifuged at 5000 × g for 5 min and transferred to a 1.5-ml centrifuge tube. Exosomes could be used for further research or stored at − 80 °C.

### Quality control of exosome isolation and verification

#### Transmission electron microscopy (TEM)

The purified exosome sample was diluted 100-fold with PBS (SH30256.01, HyClone, Logan, UT, USA) and used for electron microscopy (JEM-1400, JEOL, Akishima, Tokyo, Japan). A total of 20 μl of the diluted sample was pipetted, dropped to the center of a copper mesh, and allowed to stand for 20 min. Then, the liquid was blotted with filter paper to prepare the negative stain. Negative staining was performed with 1% phosphotungstic acid for approximately 10 s, and filter paper was used to blot the excess liquid. Then, 20 μl ddH2O was carefully added to the center of the copper mesh, and after 20 s, the remaining liquid was aspirated from the mesh with filter paper. The finished copper mesh was ready for exosome identification. TEM showed the presence of exosomes as rounded, biconcave-disk shaped, vesicle-like structures (Additional file 1: Fig. S1A).

#### Nanoparticle tracking analysis (NTA)

The extracted exosomes were diluted 1:200 (filtered PBS). The module detected by the Nano Sight 300 (Malvern Instruments Ltd., Malvern, UK) was washed with ddH2O 3 times, and then the module was purged with syringe pumping air 7–8 times to remove as much residual fluid as possible. One milliliter of the well-mixed and diluted exosome sample was aspirated into the module with a syringe, and the air bubbles in the module detection chamber were ejected. The number of images capturing repetitions was set to 3, and the duration of each capture was set to 60 s. NTA showed that the peaks of circulating exosomes from RCC, patients with benign masses, and healthy controls were 74 nm, 77 nm, and 79 nm, respectively, ranging from 50 to 200 nm (Additional file 1: Fig. S1B).

#### Western blot (WB)

A total of 50 μl RIPA lysis buffer (P0013C, Beyotime, Shanghai, China) and 0.5 μl of protease inhibitor were added to the extracted exosomes. The mixture was centrifuged at 15,000 × g for 20 min at 4 °C. The supernatant was collected, and the concentration was measured using a BCA kit (5,000,112, Bio–Rad, Hercules, CA, USA). Next, 5 × loading buffer (P0015, Beyotime, Shanghai, China) was added to the samples. Those samples were incubated in a 99 °C metal bath (Thermomixer comfort, Eppendorf, Hamburg, Germany) for 15 min. A PAGE Gel Fast Preparation Kit (PG112, EpiZyme, Shanghai, China) was used to make a 10% separating glue and 5% concentrated glue according to the manufacturer’s instructions. Then, 30-μg exosome protein samples were added to each well, and 5-μl protein marker (P0077, Beyotime, Shanghai, China) was added to the blank well. The machine (165–8029, Bio–Rad, Hercules, CA, USA) was set to 120 V, and electrophoresis was performed for 2 h. After electrophoresis was completed, and the gel was removed, stacked with a PVDF (IPFL00010, Millipore, Burlington, MA, USA) membrane, placed in a transfer tank, and filled with transfer buffer. The instrument was set to 300 mA for 2 h. After, the transfer was completed, the membranes were blocked with 5% BSA for 2 h at room temperature, and the membranes were cut at appropriate positions. Then, antibodies against CD9 (1:1000; AP1482D, ABGENT, Suzhou, Jiangsu, China), CD63 (1:500; AP5333B, ABGENT, Suzhou, Jiangsu, China), CD81 (1:4000; AM8557B, ABGENT, Suzhou, Jiangsu, China), TSG101 (1:2000; AM8662b, ABGENT, Suzhou, Jiangsu, China), and β-actin (1:5000; A5441, Sigma, St. Louis, MO, USA) were added and incubated at 4 °C overnight. The next day, the membranes were placed at room temperature for 15 min, and the primary antibody was removed. The membranes were washed 6 times with TBST for 5 min each time. Then, secondary antibodies were added, incubated for 2 h at room temperature, and washed 6 times with TBST for 5 min each time. Clarity Max Western ECL Substrate (P0018 FS, Beyotime, Shanghai, China) was added. Blots were imaged by an imaging device (e-Blot, Shanghai, China). WB showed that exosomal biomarkers, including CD9, CD63, CD81, and TSG101, were detectable in isolated exosomes (Additional file 1: Fig. S1C).

### Circulating exosomal RNA purification and sequencing

Circulating exosomal RNA was purified using an exoRNeasy Serum/Plasma Maxi Kit (217,084, Qiagen, Dusseldorf, North Rhine-Westphalia, Germany). Total RNA sequencing utilized 0.25–50 ng of RNA as the input. gDNA removal was performed by adding HL-dsDNase (70,800–202, ArcticZymes, Tromsø, Norway) and reaction buffer (66,001, ArcticZymes, Tromsø, Norway). Libraries for total RNA sequencing were prepared using SMARTer Stranded Total RNA-Seq Kit v2-Pico Input Mammalian (634,413, Clontech, Mountain View, CA, USA). Compared to the manufacturer’s protocol, the fragmentation step was set to 2 min at 94 °C; hereafter, the option to start from highly degraded RNA was followed. Library preparation also included cDNA synthesis, 5 cycles of indexing PCR, ribosomal cDNA depletion, and 9–16 cycles of enrichment PCR. Each library was measured for size with an Agilent High Sensitivity DNA Kit (5067–4626, Agilent, Santa Clara, CA, USA) and concentration with a Library Quantification Kit (638,324, Clontech, Mountain View, CA, USA). Alternatively, libraries can be quantified by a Qubit dsDNA HS kit (Q32854, Thermo Fisher Scientific, Waltham, MA, USA). Libraries were combined into an equimolar pool, which was measured for size and concentration, and libraries were sequenced using the HiSeq 2500 platform (Illumina, San Diego, CA, USA). To ensure quality, 10-Gb raw data per library was necessary.

### Sequencing data processing

The sequencing data were quality control processed by FastQC and trimmed by Trimmomatic with the following parameters: minimum length 30, sliding window 4, and required quality 15. Then, the clean data were mapped to the reference human genome (GRCh38.p13) by STAR with the Ensembl release-95 gene annotation database. Finally, we used htseq for expression quantification, and the R package Deseq2 to identify the differentially expressed genes between different groups. In order to reduce the false-positive rate of dysregulated genes, we performed multiple tests by Benjamini–Hochberg to obtain an adjusted *p* value.

### DNA gel electrophoresis

Validation of PCR products was performed using DNA gel electrophoresis. First, 60 ml TAE and 1 g solid agarose were mixed and heated until completely transparent. When warm, 6 µl nucleic acid stain was added, shaken well, and poured into the glue making rack. The comb teeth were inserted until they were completely solidified, and then the comb teeth were removed. The agarose gel was placed into the electrophoresis tank, 1 × TAE solution was added until the liquid level was over the gel, and the air bubbles in the upper sample well were discharged. The samples were loaded. The electrophoresis conditions were typically set to 120 V for 30 min. After running, an image of the gel was obtained.

### Reverse transcription and real-time quantitative polymerase chain reaction (PCR)

Circulating exosomal RNA was extracted and purified using an exoRNeasy Serum/Plasma Maxi Kit (217,084, Qiagen, Dusseldorf, North Rhine-Westphalia, Germany). RNA was reverse transcribed into cDNA according to the instructions of the PrimeScript™ RT reagent Kit (RR047A, Takara, Kyoto, Japan). The operation should be performed on ice, and the Master Mix should be prepared in an amount twice the number of reactions. Then, 10 µl should be dispensed into each reaction tube. The reverse transcription reaction conditions were as follows: 37 °C for 60 min, 85 °C for 5 s, and cooling on ice before use. The synthesized cDNA could be used immediately for subsequent qPCRs or temporarily stored at − 20 °C.

The primers and probes are presented in Additional file 2: Table S2. An ABI 7500 fluorescent PCR instrument (Applied Biosystems, Foster City, CA, USA) was used. Using 20 μl of the qPCR system, specifically 10 μl of 2 × qPCR Master Mix and 2 μl of cDNA solution, the working concentration of the upstream and downstream primers was 10 μM, the probes were used at a concentration of 5 μM, and the reaction was brought up to 20 μl with RNase-free water. The qPCR procedure was set as follows: 37 °C for 5 min, 95 °C for 10 min, and 40 cycles of 95 °C for 15 s and extension at 59 °C for 1 min.

### EmRNA quantification

We applied the standard-curve quantitation method for emRNA quantification. This method is similar to previous approaches used for other RNA types [44]. Briefly, we synthesized the amplified fragments of each target gene. During the reverse transcription of these genes, we generated a standard curve using real-time quantitative PCR by testing synthesized transcripts at different copy number concentration gradients. By this means, we could calculate the copy numbers of target genes relative to a standard sample. Specifically, an initial amount of 400 µl serum was defined to extract RNA from exosomes. Then, the extracted RNA was dissolved in RNase-free water. Then, 20 µl of emRNA was processed for reverse transcription to yield 60 µl of cDNA. Then, we added 2 µl of cDNA to 20 µl of the fluorescence PCR system, with triplicate samples accessed to yield the mean CT value. At the same time, we synthesized the target RNA transcripts at copy numbers of 10^3^, 10^4^, 10^5^, 10^6^, and 10^7^. These synthesized mRNAs were processed using the same procedure as the abovementioned transcripts (20 µl emRNA to 60 µl cDNA and 2 µl cDNA to 20 µl PCR system). These synthesized mRNAs yielded a standard curve, and we could calculate the copy number of the target emRNA by matching the CT value to the standard curve.

### Statistical analysis

Baseline analysis of the clinical characteristics of the population enrolled in this study was processed by SPSS 21.0 (IBM Corporation, Armonk, New York, USA). The measurement data were tested for normality and variance homogeneity test, and the independent samples that met the normal distribution and were tested by *t* test in group design (the *t*' test was used for variance nonhomogeneity), and the Mann–Whitney *U* test was used if they did not meet the normal distribution; paired data were tested for normality of the mean difference, using the paired *t* test for normal distribution and the Wilcoxon signed rank test for nonnormality. The chi-square test or Fisher’s exact probability test was used for the count data.

Experimental results, such as comparative analysis of gene expression, one-way analysis for graphing, and calculation of *p* value, were performed by GraphPad Prism 8 (GraphPad Software, San Diego, CA, USA). *Z*-score (*z* = (*x* − *μ*)/*σ*; *μ* = average(); *σ* = stdevp()) was applied in data pre-processing to normalize the data. MedCalc 18.0 software (MedCalc Software, Ostend, Belgium) was used to establish a clinical diagnostic model by logistic regression and calculated the area under the curve (AUC), sensitivity, and specificity. The differences were considered statistically significant at *p* < 0.05. The area under the ROC curve values ranged from 0.5 to 1, with low diagnostic value between 0.5 and 0.7, moderate diagnostic value between 0.7 and 0.9, and high diagnostic value above 0.9.

## Supplementary Information

**Additional file 1:** **Fig. S1.** Quality control ofexosome isolation and verification. **Fig.S2.** Circulating exosomal RNA screening and testing. **Fig. S3**. The performance of candidate emRNAs for screeninglocalized clear cell renal cell carcinoma (ccRCC) patients from healthycontrols and differentiating ccRCCs from patients with benign renal masses. **Fig. S4.** AUC of the signature derivedto distinguish ccRCC from healthy controls for ccRCC versus benign renal masses(AUC = 0.559).

**Additional file 2:** **Table S1**. Demographic andclinical characteristics of participants with benign solid and cystic masses. **Table S2.** List of primers and probes. **Table S3**. List of circulating exosomaldysregulated transcripts between clear cell renal cell carcinoma (ccRCC)patients and healthy controls.

**Additional file 3:** Supporting information.

## Data Availability

The clinical datasets analyzed during the current study are included in this published article and its supplementary information files, and the exosomal RNA sequencing data are available upon reasonable request to the CNGB Sequence Archive (CNSA: https://db.cngb.org/cnsa/) of CNGBdb with accession number CNP0002099.

## Abbreviations

AML: Angiomyolipoma
AUC: Area under the curve
ccRCC: Clear cell renal cell carcinoma
CT: Computed tomography
emRNA: Exosomal mRNA
exRNAs: Exosomal RNAs
MRI: Magnetic resonance imaging
NTA: Nanoparticle tracking analysis
PCR: Polymerase chain reaction
ROC: Receiver operator characteristic
SRMs: Small renal masses
TEM: Transmission electron microscopy
WB: Western blot
