# Supplementary material for: Circulating exosomal mRNA signatures for the early diagnosis of clear cell renal cell carcinoma
Source: BMC Med. 2022 Aug 25;20:270. doi: 10.1186/s12916-022-02467-1 (PMC9404613; doi:10.1186/s12916-022-02467-1)
Supplement: Supplementary file 1 — Additional file 1: Fig. S1. Quality control ofexosome isolation and verification. Fig.S2. Circulating exosomal RNA screening and testing. Fig. S3. The performance of candidate emRNAs for screeninglocalized clear cell renal cell carcinoma (ccRCC) patients from healthycontrols and differentiating ccRCCs from patients with benign renal masses. Fig. S4. AUC of the signature derivedto distinguish ccRCC from healthy controls for ccRCC versus benign renal masses(AUC = 0.559). [file 12916_2022_2467_MOESM1_ESM.zip › Figure S1 legendR3.docx]

**Fig. S1**

**Quality control of exosome isolation and verification. A,** TEM showed the presence of exosomes isolated from the serum samples of localized ccRCCs, patients with benign renal masses and healthy controls. **B,**  NTA showed the peaks of circulating exosomes from localized ccRCCs, patients with benign masses and healthy controls, the particle size distribution of exosomes isolated from the serum samples of localized ccRCCs, patients with benign renal masses and healthy controls. **C,** WB showed the exosomal markers CD9, CD63, CD81, TSG101, and β-Actin in exosomes isolated from serum samples of localized ccRCCs, patients with benign renal masses and healthy controls. TEM, transmission electron microscopy; ccRCC, clear cell renal cell carcinoma; NTA, nanoparticle tracking analysis; WB, Western blotting
