# Supplementary material for: Circulating exosomal mRNA signatures for the early diagnosis of clear cell renal cell carcinoma
Source: BMC Med. 2022 Aug 25;20:270. doi: 10.1186/s12916-022-02467-1 (PMC9404613; doi:10.1186/s12916-022-02467-1)
Supplement: Supplementary file 1 — Additional file 1: Fig. S1. Quality control ofexosome isolation and verification. Fig.S2. Circulating exosomal RNA screening and testing. Fig. S3. The performance of candidate emRNAs for screeninglocalized clear cell renal cell carcinoma (ccRCC) patients from healthycontrols and differentiating ccRCCs from patients with benign renal masses. Fig. S4. AUC of the signature derivedto distinguish ccRCC from healthy controls for ccRCC versus benign renal masses(AUC = 0.559). [file 12916_2022_2467_MOESM1_ESM.zip › Figure S3 legendR3.docx]

**Fig. S3**

**The performance of candidate emRNAs for screening localized clear cell renal cell carcinoma (RCC) patients from healthy controls and differentiating ccRCCs from patients with benign renal masses.** **A,** ROC-AUC evaluation showed the screening performance of the candidate emRNAs, including CUL9, KMT2D, PBRM1, PREX2, and SETD2, to distinguish localized ccRCCs (n=92) from healthy controls (n=50) in the training phase. **B,** ROC-AUC evaluation showed the screening performance of KMT2D and PREX2, identified as significant biomarkers for ccRCC screening by multivariate logistic regression analysis, to distinguish localized ccRCCs (n=106) from healthy controls (n=97) in the validation phase. **C,** ROC-AUC evaluation showed the diagnostic performance of CUL9, KMT2D, and PREX2, identified as significant biomarkers for ccRCC diagnostic by multivariate logistic regression analysis, to differentiate localized ccRCCs (n=106) from patients with benign renal masses (n=73). ccRCC, clear cell renal cell carcinoma; ROC, receiver operator characteristic; AUC, area under the curve
