# Supplementary material for: Circulating exosomal mRNA signatures for the early diagnosis of clear cell renal cell carcinoma
Source: BMC Med. 2022 Aug 25;20:270. doi: 10.1186/s12916-022-02467-1 (PMC9404613; doi:10.1186/s12916-022-02467-1)
Supplement: Supplementary file 1 — Additional file 1: Fig. S1. Quality control ofexosome isolation and verification. Fig.S2. Circulating exosomal RNA screening and testing. Fig. S3. The performance of candidate emRNAs for screeninglocalized clear cell renal cell carcinoma (ccRCC) patients from healthycontrols and differentiating ccRCCs from patients with benign renal masses. Fig. S4. AUC of the signature derivedto distinguish ccRCC from healthy controls for ccRCC versus benign renal masses(AUC = 0.559). [file 12916_2022_2467_MOESM1_ESM.zip › Figure S4 legendR3.docx]

**Fig. S4**

**AUC of the signature derived to distinguish ccRCC from healthy controls for ccRCC versus benign renal masses (AUC = 0.559).** ccRCC, clear cell renal cell carcinoma; AUC, area under the curve
