# Supplementary material for: Circulating exosomal mRNA signatures for the early diagnosis of clear cell renal cell carcinoma
Source: BMC Med. 2022 Aug 25;20:270. doi: 10.1186/s12916-022-02467-1 (PMC9404613; doi:10.1186/s12916-022-02467-1)
Supplement: Supplementary file 2 — Additional file 2: Table S1. Demographic andclinical characteristics of participants with benign solid and cystic masses. Table S2. List of primers and probes. Table S3. List of circulating exosomaldysregulated transcripts between clear cell renal cell carcinoma (ccRCC)patients and healthy controls. [file 12916_2022_2467_MOESM2_ESM.zip › Table S2R3.docx]

**Table S2** List of primers and probes

| NO | Gene name | Primer sequence | Product length (bp) | Probe sequence |
| --- | --- | --- | --- | --- |
| 1 | *ARID1A*-F3 | GTCAACAGGGCCAGACTCCA | 111 | vic-CAACCAGCAAAGTCCTCACCCT-mgb |
|  | *ARID1A*-R3 | ACGAAGGTTGGGCATGAGG |  |  |
| 2 | *ARID1A*-F4 | CATTTGGGTCTCAGGCATCC | 75 | / |
|  | *ARID1A*-R4 | GGCTCAGGTTCATATCTTCTTGC |  |  |
| 3 | *ATM*-F3 | CAAATGTGCAAACAGAAGAGCA | 101 | / |
|  | *ATM*-R3 | CTCCGTAAATAGCACCATTAGATGA |  |  |
| 4 | *ATM-*F63 | TGCTCAGTGTTGGTGGACAAG | 83 | vic-ATTTGCTCATACAGCAGGCCATA-mgb |
|  | *ATM-*R63 | CCATCCTGGGAAAAGTCGG |  |  |
| 5 | *CUL9*-F4 | AGCCTTTTACTCCCCACCATT | 115 | / |
|  | *CUL9*-R4 | GCAGTGTCTGCTGCACATATTCT |  |  |
| 6 | *CUL9*-F2 | CATCCTCATGTGGCTGTCGG | 89 | vic-TCCTGAGGTCTACGCCAACTGCC-mgb |
|  | *CUL9*-R2 | CGTGCTGAAGTCCCTTAGATAGTG |  |  |
| 7 | *KMT2D*-F51 | AGCACCTTCACAGGCGAGAC | 102 | vic-CAGCCGCCGGTACTGAGATGACTT-mgb |
|  | *KMT2D*-R51 | CACGTTGTTCTTCCATTCGGT |  |  |
| NO | Gene name | Primer sequence | Product length (bp) | Probe sequence |
| 8 | *KMT2D*-F31 | TCGGGCATCTCAGGTAGAGC | 99 | / |
|  | *KMT2D*-R31 | GGGTGACTTGGAGGAGAAGGT |  |  |
| 9 | *PBRM1*-F2 | AAGAGCTACCTCCCCTTCCAG | 93 | fam-CACCATTCTGTGTCAACACCA-mgb |
|  | *PBRM1*-R2 | CTCCTCCTTTTCCTGCTTGG |  |  |
| 10 | *PBRM1*-F5 | AAACTCTGGGATTTGTACCTTCG | 104 | / |
|  | *PBRM1*-R5 | ACTGTGCCCTGATTGTCTTGC |  |  |
| 11 | *PREX2*-F8 | AGAACGGCGGAAAGGTTTAA | 106 | / |
|  | *PREX2*-R8 | CAGATTTCCTTGTCTGCACATCAT |  |  |
| 12 | *PREX2*-F38 | CGCTGGAGCAAGCCATCAT | 88 | vic-CTGGCCAGAAGCCACGGACT-mgb |
|  | *PREX2*-R38 | CCCTGCTTCCGCATCACAT |  |  |
| 13 | *SETD2*-F7 | GCTGTGAACCAAATTGTGAAACC | 97 | fam-ACCCAACCCTCAGTTGTCCGTTC-mgb |
|  | *SETD2*-R7 | AACTCTGAGCCTGAAGGAACCA |  |  |
| 14 | *SETD2*-F14 | CAACGGGAGGCTCAGAAACA | 113 | / |
|  | *SETD2*-R14 | GGGTAACCAGCAAAGGGATG |  |  |
