# Supplementary material for: Circulating exosomal mRNA signatures for the early diagnosis of clear cell renal cell carcinoma
Source: BMC Med. 2022 Aug 25;20:270. doi: 10.1186/s12916-022-02467-1 (PMC9404613; doi:10.1186/s12916-022-02467-1)
Supplement: Supplementary file 2 — Additional file 2: Table S1. Demographic andclinical characteristics of participants with benign solid and cystic masses. Table S2. List of primers and probes. Table S3. List of circulating exosomaldysregulated transcripts between clear cell renal cell carcinoma (ccRCC)patients and healthy controls. [file 12916_2022_2467_MOESM2_ESM.zip › Table S3R3.docx]

**Table S3** List of circulating exosomal dysregulated transcripts between clear cell renal cell carcinoma (ccRCC) patients and healthy controls

| transcript id | gene name | log2FoldChange | *p* value |
| --- | --- | --- | --- |
| ENST00000261425 | KLHL5 | 6.991977406 | 1.92E-04 |
| ENST00000586195 | EML2 | 5.215581273 | 9.49E-05 |
| ENST00000243347 | TNFAIP6 | 5.065560173 | 1.83E-04 |
| ENST00000460870 | PLXNA2 | 5.03151828 | 1.75E-04 |
| ENST00000547345 | DCTN2 | 4.967836808 | 0.001502518 |
| ENST00000556803 | SLC22A17 | 4.043252531 | 8.93E-04 |
| ENST00000463205 | TMEM237 | 3.885759016 | 7.70E-05 |
| ENST00000619181 | CLCNKB | 3.738416449 | 3.38E-04 |
| ENST00000368777 | LCE4A | 3.719697364 | 0.002215617 |
| ENST00000506275 | ITGA1 | 3.560878173 | 0.001714381 |
| ENST00000278616 | ATM | 3.550741419 | 3.69E-04 |
| ENST00000409792 | SETD2 | 3.498461493 | 1.36E-06 |
| ENST00000540040 | MTRNR2L1 | 3.447962906 | 1.09E-04 |
| ENST00000418040 | BPGM | 3.419156068 | 0.001980698 |
| ENST00000529111 | DLG2 | 3.38357362 | 0.001924745 |
| ENST00000460704 | SETD4 | 3.311278302 | 0.001567549 |
| ENST00000568875 | SNAPC5 | 3.286352712 | 0.00191997 |
| ENST00000252050 | CUL9 | 3.285334221 | 2.82E-04 |
| ENST00000588651 | ZNF627 | 3.249137058 | 0.00326428 |
| ENST00000484171 | 1-Dec | 3.040286843 | 4.27E-04 |
| ENST00000522676 | STPG2 | 3.028325669 | 2.03E-04 |
| ENST00000483468 | REPS1 | 2.98873856 | 6.65E-04 |
| ENST00000301067 | KMT2D | 2.987146908 | 4.31E-04 |
| ENST00000521389 | RNF130 | 2.865333724 | 0.001868161 |
| ENST00000544216 | LSM14A | 2.662860908 | 0.007208803 |
| ENST00000604714 | WDR11 | 2.612767925 | 2.85E-04 |
| ENST00000219439 | HSDL1 | 2.592759984 | 0.002765683 |
| ENST00000462804 | EVI2A | 2.503366605 | 0.001748672 |
| ENST00000299665 | CLEC4D | 2.420053745 | 3.37E-04 |
| ENST00000641792 | OR4E1 | 2.40648395 | 9.65E-04 |
| ENST00000504945 | ACSF2 | 2.364882007 | 0.005537689 |
| ENST00000474223 | SAT1 | 2.347689437 | 0.008378408 |
| ENST00000322354 | SERTAD3 | 2.338573216 | 0.002056661 |
| ENST00000512584 | TDO2 | 2.250634837 | 0.00488074 |
| ENST00000608868 | SMIM8 | 2.24249968 | 0.002781585 |
| ENST00000589330 | ME2 | 2.238939315 | 0.001346724 |
| ENST00000645950 | CD99 | 2.233423552 | 0.009043198 |
| ENST00000374005 | FGR | 2.211099157 | 0.005965809 |
| ENST00000518813 | PIK3R1 | 2.191821981 | 0.001046537 |
| ENST00000643453 | ATXN7 | 2.18571554 | 0.00158134 |
| ENST00000486536 | USP41 | 2.17264638 | 0.002110422 |
| ENST00000310836 | UGT8 | 2.162394206 | 0.001576988 |
| ENST00000467485 | BTN2A2 | 2.134904886 | 0.001289839 |
| ENST00000489955 | COBLL1 | 2.071931194 | 0.002183513 |
| ENST00000408045 | THADA | 2.05368243 | 0.006993829 |
| ENST00000495510 | SPART | 2.034531549 | 0.002062634 |
| ENST00000255224 | SYT4 | 2.026232491 | 0.004885641 |
| ENST00000261250 | C12orf4 | 2.024365183 | 0.004391413 |
| ENST00000491507 | ZNF717 | 2.015614272 | 0.001464003 |
| ENST00000261369 | SNX24 | 2.008809579 | 0.006545649 |
| ENST00000382760 | UPB1 | 1.991223265 | 6.04E-04 |
| ENST00000461408 | ADAM10 | 1.982089055 | 3.09E-04 |
| ENST00000263559 | VPS26A | 1.974221996 | 4.93E-04 |
| ENST00000466830 | CMC1 | 1.972611493 | 0.006102704 |
| ENST00000337537 | PPP2R5E | 1.970404971 | 4.45E-05 |
| ENST00000343600 | MBNL2 | 1.968874546 | 0.005253483 |
| ENST00000356770 | PBRM1 | 1.928030568 | 1.85E-04 |
| ENST00000351500 | PFDN5 | 1.924341111 | 0.003140336 |
| ENST00000637249 | MAGI2 | 1.924217259 | 0.002284689 |
| ENST00000360351 | MAP2 | 1.887860837 | 0.008274422 |
| ENST00000534183 | ASRGL1 | 1.882588222 | 0.00283016 |
| ENST00000488957 | ATP13A5 | 1.86875172 | 0.001633305 |
| ENST00000574814 | C19orf84 | 1.835045799 | 0.006907821 |
| ENST00000478436 | PLOD2 | 1.830857114 | 0.009353273 |
| ENST00000491448 | GTDC1 | 1.813630953 | 0.003870791 |
| ENST00000547153 | TCP11L2 | 1.78259701 | 0.001772378 |
| ENST00000593592 | IGFL2 | 1.773926247 | 4.57E-04 |
| ENST00000370521 | PKN2 | 1.771140282 | 0.002075509 |
| ENST00000538669 | CEMIP2 | 1.768783952 | 0.001680749 |
| ENST00000495461 | SELENOK | 1.768508886 | 0.003581979 |
| ENST00000356286 | RBCK1 | 1.704038459 | 4.66E-04 |
| ENST00000497003 | PDE11A | 1.679015278 | 0.001820305 |
| ENST00000369287 | CCDC186 | 1.628810796 | 0.006246362 |
| ENST00000354371 | COPS8 | 1.616563103 | 0.007487409 |
| ENST00000256474 | VHL | 1.612935553 | 4.67E-04 |
| ENST00000324856 | ARID1A | 1.608276249 | 4.72E-04 |
| ENST00000543571 | LATS1 | 1.593798456 | 0.001090182 |
| ENST00000524823 | AC138969.1 | 1.583638789 | 0.001295432 |
| ENST00000495071 | SUPT20H | 1.563812846 | 0.006216177 |
| ENST00000484162 | SRSF11 | 1.560893382 | 0.007879771 |
| ENST00000618538 | SEC22B | 1.557470569 | 1.15E-04 |
| ENST00000532628 | TSPAN19 | 1.516340884 | 0.00469332 |
| ENST00000635641 | AL031777.3 | 1.500433476 | 0.006497651 |
| ENST00000647020 | HBB | 1.497621219 | 0.042329537 |
| ENST00000578795 | BLMH | 1.486507316 | 0.002303406 |
| ENST00000366675 | TAF5L | 1.478824104 | 2.64E-04 |
| ENST00000513840 | ZNF827 | 1.478336164 | 0.010422926 |
| ENST00000381620 | GABRA2 | 1.460245569 | 0.009822172 |
| ENST00000256785 | CFHR5 | 1.433085372 | 0.003096329 |
| ENST00000640957 | SCARB2 | 1.4220421 | 0.001936914 |
| ENST00000483726 | UBN2 | 1.410212333 | 0.006036378 |
| ENST00000271450 | FCGR2A | 1.390346804 | 0.001619384 |
| ENST00000274278 | UGT3A1 | 1.35384332 | 0.005342213 |
| ENST00000636106 | TTC33 | 1.347295233 | 0.007358172 |
| ENST00000396005 | SLC5A12 | 1.344693824 | 0.001781772 |
| ENST00000548783 | CSRP2 | 1.342935104 | 0.002791059 |
| ENST00000360280 | VPS13A | 1.33855359 | 0.005837402 |
| ENST00000541363 | HMGA2 | 1.33567333 | 8.21E-04 |
| ENST00000256951 | EMP1 | 1.333412503 | 8.24E-04 |
| ENST00000294649 | NTNG1 | 1.331912672 | 0.005647683 |
| ENST00000551483 | CNOT2 | 1.321597761 | 0.004338553 |
| ENST00000636502 | REELD1 | 1.305635505 | 0.006103914 |
| ENST00000484479 | DACH2 | 1.300734905 | 0.006708425 |
| ENST00000264318 | GABRA4 | 1.293730627 | 0.002424019 |
| ENST00000454520 | RACGAP1 | 1.286440269 | 0.002657244 |
| ENST00000638165 | NFIB | 1.283630714 | 0.00466177 |
| ENST00000382103 | SEPSECS | 1.278333785 | 0.003393909 |
| ENST00000324439 | RCHY1 | 1.261865118 | 0.004725733 |
| ENST00000296420 | EMCN | 1.252754507 | 0.007388816 |
| ENST00000455502 | GNGT1 | 1.22577112 | 0.007083847 |
| ENST00000571204 | ACSM2A | 1.218660945 | 0.00106664 |
| ENST00000491010 | TBRG1 | 1.204456117 | 0.005808894 |
| ENST00000637622 | CACNB4 | 1.181280451 | 0.003014327 |
| ENST00000470802 | ROBO2 | 1.177990258 | 0.004809907 |
| ENST00000604238 | MICU1 | 1.167318843 | 0.002322273 |
| ENST00000430262 | ZNF652 | 1.161731788 | 0.003824953 |
| ENST00000568344 | SYT16 | 1.158716234 | 0.001972424 |
| ENST00000445816 | SERF2 | 1.155784411 | 0.002960774 |
| ENST00000483683 | COL4A2 | 1.132089981 | 0.005885819 |
| ENST00000288368 | PREX2 | 1.1134523 | 4.21E-04 |
| ENST00000370489 | ENTPD7 | 1.107149099 | 0.001295468 |
| ENST00000308666 | ABCD2 | 1.104003213 | 0.00134573 |
| ENST00000264977 | PPP2R3A | 1.098516417 | 0.001046242 |
| ENST00000510312 | ARHGEF28 | 1.098456211 | 0.00383954 |
| ENST00000306858 | FAM83B | 1.098347283 | 0.001656157 |
| ENST00000360726 | PAQR8 | 1.095485649 | 0.003126084 |
| ENST00000619158 | CDH11 | 1.088129007 | 0.005844463 |
| ENST00000372769 | SEMG2 | 1.080581648 | 0.001402929 |
| ENST00000452556 | SPAG16 | 1.079644971 | 0.002943301 |
| ENST00000261758 | MESD | 1.064808885 | 0.002141297 |
| ENST00000465868 | ANKRD30BL | 1.061970126 | 0.005474365 |
| ENST00000476722 | SDCCAG8 | 1.048532609 | 0.010605814 |
| ENST00000373930 | MEGF9 | 1.047647276 | 0.00348105 |
| ENST00000263960 | COQ10B | 1.02916005 | 0.002227265 |
| ENST00000371321 | CYP2C19 | 1.021017117 | 0.002698097 |
| ENST00000546444 | ARHGAP29 | 1.020195254 | 0.001409502 |
| ENST00000302103 | FUT9 | 1.006702782 | 0.003500123 |
| ENST00000602295 | ANKRD18A | 1.005785643 | 0.00301672 |
| ENST00000309964 | CLOCK | 1.005714569 | 0.006267192 |
| ENST00000356454 | SOWAHC | -1.003782146 | 0.002334764 |
| ENST00000642298 | AC104389.4 | -1.017741252 | 0.012371063 |
| ENST00000534751 | PRDM11 | -1.027265285 | 0.00223635 |
| ENST00000411432 | METTL21A | -1.088434153 | 0.014803904 |
| ENST00000576995 | BAIAP2 | -1.091716359 | 0.001219195 |
| ENST00000370220 | LZTS2 | -1.112689073 | 0.002350602 |
| ENST00000421177 | NSG1 | -1.131196073 | 0.041163408 |
| ENST00000388820 | ADAMTS7 | -1.198619029 | 0.04775868 |
| ENST00000377707 | SHB | -1.204521572 | 0.001991026 |
| ENST00000303991 | GPRIN1 | -1.234697458 | 0.001646582 |
| ENST00000309415 | SH3RF3 | -1.281132992 | 7.11E-04 |
| ENST00000622824 | B3GNT6 | -1.301990954 | 0.001633282 |
| ENST00000342665 | SOX12 | -1.313171787 | 0.041980391 |
| ENST00000262450 | CHD5 | -1.329972851 | 6.64E-04 |
| ENST00000567216 | CAPN15 | -1.332493871 | 0.001927157 |
| ENST00000328449 | C1QTNF8 | -1.431199501 | 0.001045537 |
| ENST00000382252 | ZNF316 | -1.449403463 | 4.48E-04 |
| ENST00000568223 | METRN | -1.474004262 | 0.033177464 |
| ENST00000610774 | ARFRP1 | -1.492970861 | 6.36E-04 |
| ENST00000401095 | TTC34 | -1.519288393 | 9.58E-05 |
| ENST00000382349 | ONECUT3 | -1.592329366 | 1.29E-04 |
| ENST00000568546 | TBL3 | -1.630908577 | 8.82E-05 |
| ENST00000556755 | TBPL2 | -1.733780273 | 0.001354669 |
| ENST00000599531 | PNMA8B | -1.772257309 | 2.22E-04 |
| ENST00000402696 | TF | -1.774631346 | 0.018910615 |
| ENST00000377893 | GPR153 | -1.796831819 | 2.24E-04 |
| ENST00000173229 | NTN1 | -2.0981221 | 0.00409645 |
| ENST00000624676 | MAFF | -2.114448769 | 0.006225208 |
| ENST00000636330 | ESPN | -2.123582679 | 0.018560224 |
| ENST00000504880 | SLC38A9 | -2.161473422 | 0.005091537 |
| ENST00000288840 | SMAD6 | -2.185082684 | 0.042037876 |
| ENST00000580279 | GDF10 | -2.207598759 | 0.01810693 |
| ENST00000457543 | ZNF853 | -2.214076728 | 0.003625731 |
| ENST00000056233 | NFE2L3 | -2.326089872 | 0.001255034 |
| ENST00000298743 | GAS1 | -2.370689188 | 0.014168405 |
| ENST00000335071 | FOXE3 | -2.411295017 | 0.014791029 |
| ENST00000373012 | POU3F1 | -2.481048905 | 0.007451559 |
| ENST00000539664 | TRIL | -2.500964555 | 1.17E-04 |
| ENST00000537909 | CDH22 | -2.519193106 | 0.00224931 |
| ENST00000307169 | INSM2 | -2.57358139 | 0.001285818 |
| ENST00000378075 | LRRC10B | -2.61065329 | 0.002537186 |
| ENST00000302006 | IRX1 | -2.618978357 | 0.001101866 |
| ENST00000332947 | FAM43B | -2.671353585 | 5.35E-04 |
| ENST00000317811 | FJX1 | -2.7101576 | 0.002028233 |
| ENST00000252723 | EPO | -2.773156185 | 2.82E-04 |
| ENST00000331289 | ASCL2 | -2.816573337 | 5.21E-04 |
| ENST00000296350 | MELTF | -2.833201758 | 3.22E-04 |
| ENST00000466324 | NRXN2 | -2.898723988 | 0.001388864 |
| ENST00000296921 | TLX3 | -2.916901471 | 0.00487316 |
| ENST00000375123 | FOXE1 | -2.976395769 | 8.53E-04 |
| ENST00000335099 | RTN4RL2 | -2.995641739 | 7.36E-05 |
| ENST00000322241 | GPR62 | -2.998490903 | 2.83E-04 |
| ENST00000234389 | GRIN3B | -3.120890155 | 1.78E-04 |
| ENST00000394967 | SP9 | -3.179254933 | 0.002592023 |
| ENST00000265340 | PITX1 | -3.186868373 | 4.98E-04 |
| ENST00000645831 | FOXC1 | -3.203470281 | 0.013180212 |
| ENST00000645481 | FOXF2 | -3.203885654 | 8.71E-04 |
| ENST00000344575 | FZD9 | -3.23033917 | 1.41E-04 |
| ENST00000291442 | NR2F6 | -3.269790185 | 4.70E-04 |
| ENST00000586582 | SEMA6B | -3.284143805 | 3.13E-05 |
| ENST00000293190 | GRIN2C | -3.32419477 | 6.23E-04 |
| ENST00000340356 | SOX18 | -3.337228691 | 8.68E-07 |
| ENST00000541682 | HES7 | -3.381983923 | 9.72E-06 |
| ENST00000378693 | SOWAHA | -3.39696436 | 9.61E-05 |
| ENST00000371116 | FOXD3 | -3.450501693 | 5.36E-04 |
| ENST00000397899 | KIAA1211L | -3.494514473 | 9.38E-06 |
| ENST00000372441 | LRRC73 | -3.507551689 | 6.05E-04 |
| ENST00000370536 | SOX3 | -3.535457091 | 0.001697024 |
| ENST00000313135 | UTS2R | -3.629118816 | 2.81E-05 |
| ENST00000585527 | LINGO3 | -3.734426044 | 2.05E-04 |
| ENST00000593649 | KANK3 | -3.738603192 | 2.68E-04 |

ccRCC, clear cell renal cell carcinoma
