# Supplementary material for: Circulating exosomal mRNA signatures for the early diagnosis of clear cell renal cell carcinoma
Source: BMC Med. 2022 Aug 25;20:270. doi: 10.1186/s12916-022-02467-1 (PMC9404613; doi:10.1186/s12916-022-02467-1)
Supplement: Supplementary file 3 — Additional file 3: Supporting information. [file 12916_2022_2467_MOESM3_ESM.docx]

**Supporting information**

**Selecting candidate emRNAs**

We first investigated circulating emRNA profiling in ccRCCs (n = 12) and healthy controls (n = 22) by RNA-seq. Two hundred and ten dysregulated (*p* < 0.05, fold change > 2 or < 0.5, FDR < 0.05) emRNAs in ccRCC were identified. The full list of 210 dysregulated emRNAs was included in the paper as Table S3.

Based on the previous reports, we selected candidate biomarkers from the list of 210 dysregulated emRNAs. After evaluating the correlation between these mRNAs and renal cancer or/and multiple malignant tumors by reviewing previous publications, we selected seven top upregulated emRNAs as candidate biomarkers for further study, including CUL9(1), ATM(2), ARID1A(3-7), KMT2D(8, 9), PBRM1(7, 10-14), PREX2(15), and SETD2(7, 10, 11, 16). Besides, the source of circulating emRNA is largely unknown, we believe that these 7 emRNAs related to ccRCC or/and multiple malignant tumors have the potential to become biomarkers for ccRCC detection. More importantly, we used these 7 emRNAs to establish a diagnostic model, and the AUC was 100% (see the blow figure), indicating these 7 emRNAs are sufficient to qualify as effective biomarkers for the detection of ccRCC. Based on these strategies, we finally selected these 7 emRNAs as candidate biomarkers for further testing.


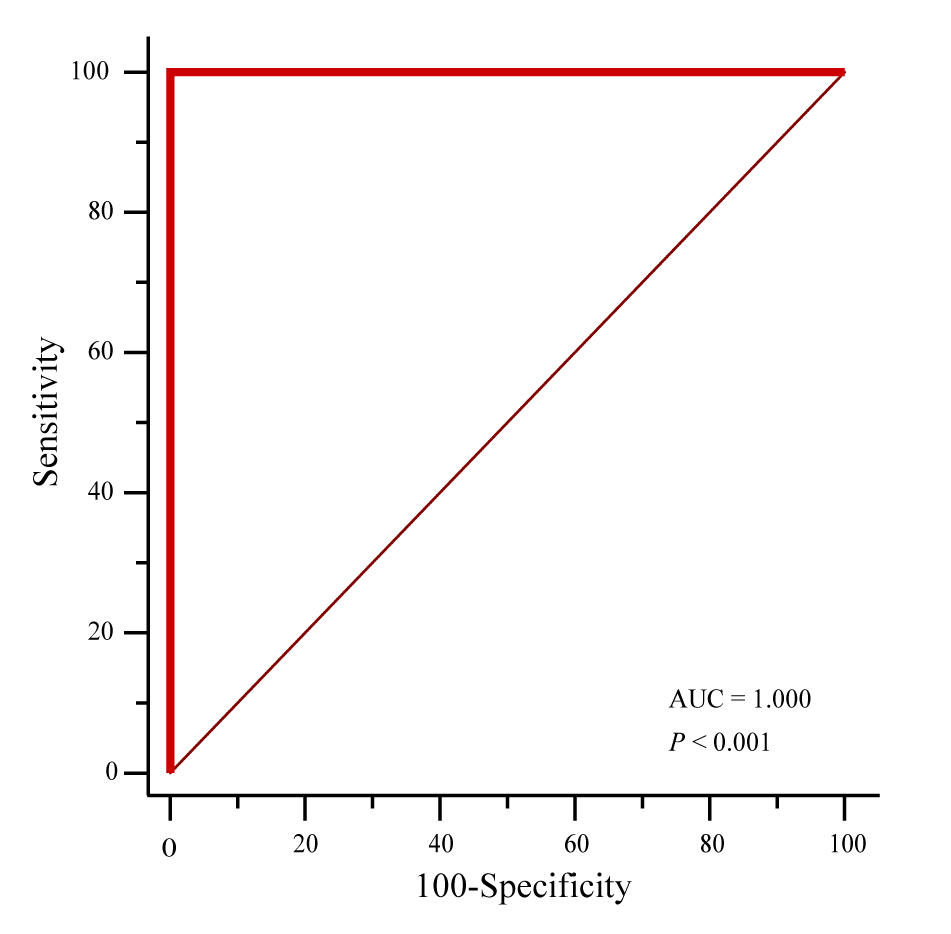


AUC of RCC diagnostic signature for RCC versus Healthy = 1 (95% CI, 0.897 to 1).

Logistic regression model (Method of Stepwise) = 101.33592 × ARID1A + 36.46114 × ATM +38.14562 × CUL9 - 11.78465 × KMT2D + 79.15301 × PBRM1 +33.27988 × PREX2 + 19.35630 × SETD2 - 65.11576.

**References**

1. Cianflone F, Lazarevic D, Palmisano A, Fallara G, Larcher A, Freschi M, et al. Radiomic and gEnomic approaches for the enhanced DIagnosis of clear cell REnal Cancer (REDIRECt): a translational pilot methodological study. Translational andrology and urology. 2022;11(2):149-58.

2. Ren W, Xue B, Chen M, Liu L, Zu X. Low Expression of ATM Indicates a Poor Prognosis in Clear Cell Renal Cell Carcinoma. Clin Genitourin Cancer. 2019;17(3):e433-e9.

3. Somsuan K, Peerapen P, Boonmark W, Plumworasawat S, Samol R, Sakulsak N, et al. ARID1A knockdown triggers epithelial-mesenchymal transition and carcinogenesis features of renal cells: role in renal cell carcinoma. FASEB journal : official publication of the Federation of American Societies for Experimental Biology. 2019;33(11):12226-39.

4. Xiao W, Lou N, Ruan H, Bao L, Xiong Z, Yuan C, et al. Mir-144-3p Promotes Cell Proliferation, Metastasis, Sunitinib Resistance in Clear Cell Renal Cell Carcinoma by Downregulating ARID1A. Cellular physiology and biochemistry : international journal of experimental cellular physiology, biochemistry, and pharmacology. 2017;43(6):2420-33.

5. Park JH, Lee C, Suh JH, Chae JY, Kim HW, Moon KC. Decreased ARID1A expression correlates with poor prognosis of clear cell renal cell carcinoma. Human pathology. 2015;46(3):454-60.

6. Lichner Z, Scorilas A, White NM, Girgis AH, Rotstein L, Wiegand KC, et al. The chromatin remodeling gene ARID1A is a new prognostic marker in clear cell renal cell carcinoma. Am J Pathol. 2013;182(4):1163-70.

7. Cancer Genome Atlas Research N. Comprehensive molecular characterization of clear cell renal cell carcinoma. Nature. 2013;499(7456):43-9.

8. Zhu J, Liu Z, Liang X, Wang L, Wu D, Mao W, et al. A Pan-Cancer Study of KMT2 Family as Therapeutic Targets in Cancer. Journal of oncology. 2022;2022:3982226.

9. Chen G, Chen P, Zhou J, Luo G. Pan-cancer analysis of histone methyltransferase KMT2D with potential implications for prognosis and immunotherapy in human cancer. Combinatorial chemistry & high throughput screening. 2022.

10. Suh J, Jeong C, Choi S, Ku J, Kim H, Kim K, et al. Sharing the initial experience of pan-cancer panel analysis in high-risk renal cell carcinoma in the Korean population. BMC urology. 2020;20(1):125.

11. Park JS, Lee HJ, Cho NH, Kim J, Jang WS, Heo JE, et al. Risk Prediction Tool for Aggressive Tumors in Clinical T1 Stage Clear Cell Renal Cell Carcinoma Using Molecular Biomarkers. Computational and structural biotechnology journal. 2019;17:371-7.

12. Guvenis A, Ökmen HB, Uysal H. Predicting the polybromo-1 (PBRM1) mutation of a clear cell renal cell carcinoma using computed tomography images and KNN classification with random subspace. Vibroengineering PROCEDIA. 2019;26:30-4.

13. Wang Z, Peng S, Guo L, Xie H, Wang A, Shang Z, et al. Prognostic and clinicopathological value of PBRM1 expression in renal cell carcinoma. Clinica chimica acta; international journal of clinical chemistry. 2018;486:9-17.

14. Espana-Agusti J, Warren A, Chew SK, Adams DJ, Matakidou A. Loss of PBRM1 rescues VHL dependent replication stress to promote renal carcinogenesis. Nature communications. 2017;8(1):2026.

15. Barrows D, Schoenfeld S, Hodakoski C, Silkov A, Honig B, Couvillon A, et al. p21-activated Kinases (PAKs) Mediate the Phosphorylation of PREX2 Protein to Initiate Feedback Inhibition of Rac1 GTPase. The Journal of biological chemistry. 2015;290(48):28915-31.

16. Santos VE, da Costa WH, Bezerra SM, da Cunha IW, Nobre JQC, Brazão ES, Jr., et al. Prognostic Impact of Loss of SETD2 in Clear Cell Renal Cell Carcinoma. Clin Genitourin Cancer. 2021;19(4):339-45.
